# Deubiquitinase USP9x regulates the proline biosynthesis pathway in non-small cell lung cancer

**DOI:** 10.1038/s41420-024-02111-2

**Published:** 2024-07-29

**Authors:** Tina Becirovic, Boxi Zhang, Cecilia Lindskog, Erik Norberg, Helin Vakifahmetoglu-Norberg, Vitaliy O. Kaminskyy, Elena Kochetkova

**Affiliations:** 1 Department of Physiology and Pharmacology, Solnavägen 9, Biomedicum, Karolinska Institutet, 171 65 Stockholm, Sweden; 2 Department of Immunology, Genetics and Pathology, Rudbeck Laboratory, Uppsala University, 751 85 Uppsala, Sweden

**Keywords:** Cancer metabolism, Non-small-cell lung cancer, Ubiquitylated proteins

## Abstract

Metabolic rewiring has been recognized as a hallmark of malignant transformation, supplying the biosynthetic and energetic demands for rapid cancer cell proliferation and tumor progression. A comprehensive understanding of the regulatory mechanisms governing these metabolic processes is still limited. Here, we identify the deubiquitinase ubiquitin-specific peptidase 9 X-linked (USP9x) as a positive regulator of the proline biosynthesis pathway in non-small cell lung cancer (NSCLC). Our findings demonstrate USP9x directly stabilizes pyrroline-5-carboxylate reductase 3 (PYCR3), a key enzyme in the proline cycle. Disruption of proline biosynthesis by either USP9x or PYCR3 knockdown influences the proline cycle leading to a decreased activity of the connected pentose phosphate pathway and mitochondrial respiration. We show that USP9x is elevated in human cancer tissues and its suppression impairs NSCLC growth in vitro and in vivo. Overall, our study uncovers a novel function of USP9x as a regulator of the proline biosynthesis pathway, which impacts lung cancer growth and progression, and implicates a new potential therapeutic avenue.

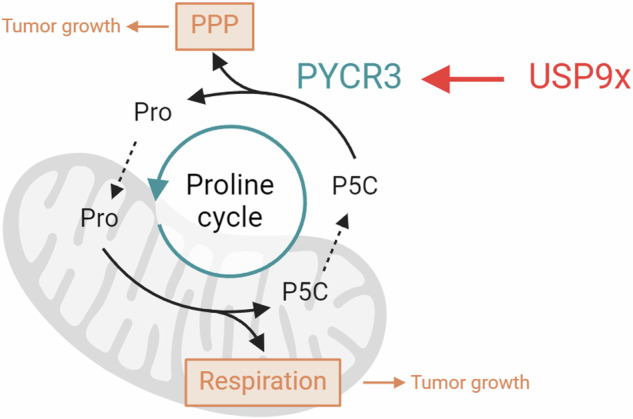

## Introduction

Alterations in metabolic pathways promoting tumor growth represent a hallmark of cancer [1]. Mechanistic insights into how the oncogenotype dictates metabolic patterns are well-understood and microenvironmental as well as genetic factors also appear to induce a selective pressure that drives clonal evolution within tumors, which can generate or eliminate metabolic dependencies [2, 3]. However, we still possess a limited understanding of the processes governing tumor metabolism to identify vulnerabilities suitable for an efficient therapeutic targeting in vivo.

Lung cancer remains the leading cause of cancer-related deaths worldwide [4] with non-small cell lung cancer (NSCLC) being the most common histological subtype. There is a great need for understanding the underlying pathomechanisms of NSCLC and for defining disease-specific targets that would pave the way for the development of more effective therapies. During recent years, targeting metabolic addictions has been proposed as an emerging approach [5]. In fact, it is well documented that lung cancer cells tend to shift their metabolic activities towards glycolysis, also known as the Warburg effect, to provide precursors required for their rapid proliferation [6]. Increasing evidence further suggests that metabolic rearrangements in NSCLC not only occur in the pathways of central carbon metabolism, but also in other conduits, such as in serine biosynthesis [7] or lipid metabolism [8]. Yet, defining a therapeutic window for targeting metabolic vulnerabilities differing between proliferating cancer and normal cells remains challenging. Therefore, uncovering tumor-specific mechanisms of metabolic regulation emerges as a desirable promising therapeutic avenue.

Protein stability controlled by the ubiquitin proteasome system (UPS) can govern the abundance of proteins present in the cell by targeting polyubiquitinated proteins for recognition and processing in the proteasome. Ubiquitin molecules are attached to a target protein by the enzymatic activities of E1 activating, E2 conjugating and E3 ubiquitin ligases and are recognized by ubiquitin receptors [9]. In contrast, deubiquitinases (DUBs) have the opposing function of removing monoubiquitin or polyubiquitin chains from the target proteins [10]. The balanced interplay of these two enzyme families coordinates ubiquitin tagging of proteins, ensuring a fine management of protein turnover, thereby maintaining optimally functioning proteins [11]. Given their fundamental role in regulating protein stability and activity, it comes to no surprise that dysregulation of DUBs has been linked to most cancer-related pathways, both as tumor promoters and tumor suppressors [12]. Numerous DUBs have been identified to directly contribute to cancer cell proliferation. For instance, OTUD7B deubiquitinates key cell cycle regulators [13] and USP2 was shown to stabilize cyclin D1, a known proto-oncogene, causing an aberrant cell cycle progression in many different tumor types [14]. Furthermore, several DUBs, including HAUSP/USP7, USP10, USP11, USP13, OTUD3, and Ataxin-3 have been shown to reverse the ubiquitination of PTEN, a key antagonist in the PI3K growth-promoting pathway, implicating its function in cancer-specific contexts [15]. While exploration of therapeutic targeting of DUBs is an increasing area of research, most DUBs are poorly understood when it comes to their exact function, targets, and regulation. Yet, due to their well-defined catalytic sites, DUBs represent suitable targets for small-molecule drug discovery [16].

Recently, stabilization of metabolic enzymes through removal of ubiquitin has been proposed as one of the mechanisms by which cancer cells enhance their metabolic rate. Currently, only few DUBs out of the >90 DUBs encoded in the human genome, including OTUB2, USP13, and JOSD2, have been attributed to display such function [17–19]. Whether additional DUBs can control protein stability relevant for metabolic rewiring in cancer cells remains undiscovered.

Here, we applied a systematic approach on all human DUBs in the context of lung cancer metabolism, starting with a broad in silico analysis to narrow down to a DUB, ubiquitin-specific peptidase 9 X-linked (USP9x), with a potential role in regulating tumor growth via the proline metabolism pathway both in vitro and in vivo. Our findings suggest a novel mechanism of proline biosynthesis regulation through direct stabilization of PYCR3 by USP9x.

## Results

### DUBs associated with tumor metabolism are elevated in human lung cancer

**Fig. 1:**
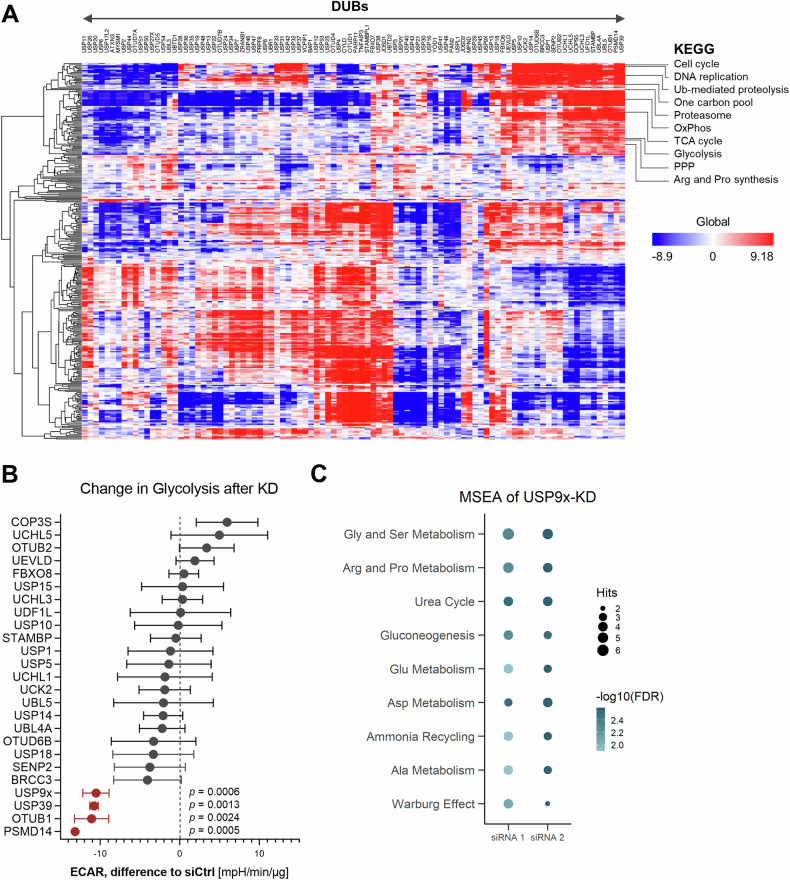
Identification of DUBs associated with metabolism. **A** Guilt-by-association analysis of all human DUBs. DUBs were arranged by similarity (Spearman correlation) in columns, GSEA of RNA expression data from the TCGA were clustered in rows. Blue: anti-correlated genes; red: correlated genes. **B** Difference in glycolysis measured by ECAR upon genetic silencing of the indicated DUB compared to the control. Red: highlights DUBs with significantly reduced glycolysis. Error bars ± SD. **C** MSEA on metabolomics data acquired from USP9x knockdown cancer cell samples. Significant pathways with at least 4 hits are depicted in the graph.

To identify DUBs that are potentially involved in tuning lung cancer metabolism, providing cells with elevated proliferation and growth ability, an unbiased analysis was employed. We hypothesized that excessive DUB expression may be positively linked with growth-promoting metabolic pathways. Therefore, a guilt-by-association analysis on human DUB gene expression data downloaded from The Cancer Genome Atlas (TCGA) public database (https://portal.gdc.cancer.gov/) was performed. All 99 DUBs were ranked according to similarity using Spearman correlation followed by gene set enrichment analysis (GSEA) of the Kyoto Encyclopedia of Genes and Genomes (KEGG) pathways. This approach identified a cluster of 25 DUBs enriched for KEGG terms associated with metabolic and growth-related activities, such as cell cycle, DNA replication, glycolysis, TCA cycle, pentose phosphate pathway, and arginine and proline synthesis (Fig. 1A), predicting that this set of DUBs may display a functional role in lung cancer metabolism and growth. Given that glycolysis is commonly dysregulated in lung cancer [20], it was examined if the DUBs within the identified cluster displayed any effect on this metabolic pathway. Therefore, siRNA-mediated knockdown was performed for each of these DUBs and glycolysis was measured in real-time in intact cells using an extracellular flux analyzer (Fig. 1B). Four DUBs (USP39, USP9x, OTUB1, and PSMD14) were identified, in which the knockdown resulted in a significant reduction of glycolysis compared to the non-targeting siRNA control (*p*-values from 0.0005 – 0.0024).

Notably, these four DUBs have been suggested to play roles in cancer progression through promoting migration and metastasis [21–24]. However, as PSMD14 is a constituent of the 26 S proteasome, it is expected to impact general protein homeostasis affecting metabolic pathways [25], OTUB1 was earlier reported to be directly linked to the regulation of the rate-limiting glycolytic enzyme, hexokinase 2 (HK2), [26], likely explaining the effect observed by its knockdown on glycolysis. While USP39 is largely identified to participate in alternative splicing [27], the precise role of USP9x in different types of cancer, in particular lung cancer, is less studied. To date, no direct metabolic enzymes have been identified as its targets. Therefore, we decided to continue our investigation on USP9x and its potential role in cellular metabolism.

To identify the metabolic pathways affected by the activity of USP9x, metabolite levels were measured using targeted capillary electrophoresis time-of-flight/mass spectrometry (CE-TOF/MS) in USP9x-depleted lung cancer cells. Metabolite set enrichment analysis (MSEA) uncovered metabolic pathways altered specifically in the USP9x-knockdown conditions compared to the control siRNA (Supplementary Table 1). The depletion of USP9x by two independent siRNAs led to significant changes in numerous pathways (Fig. 1C and Supplementary Fig. 1A). Metabolic changes with at least 4 hits were observed in 9 pathways, primarily covering metabolites associated with amino acid metabolism, including glycine and serine, arginine and proline, aspartate, alanine, glutamate, and the urea cycle. Collectively, these data suggest a potential role of USP9x as a positive regulator of metabolism in lung cancer cells.

### USP9x regulates lung tumor growth

**Fig. 2:**
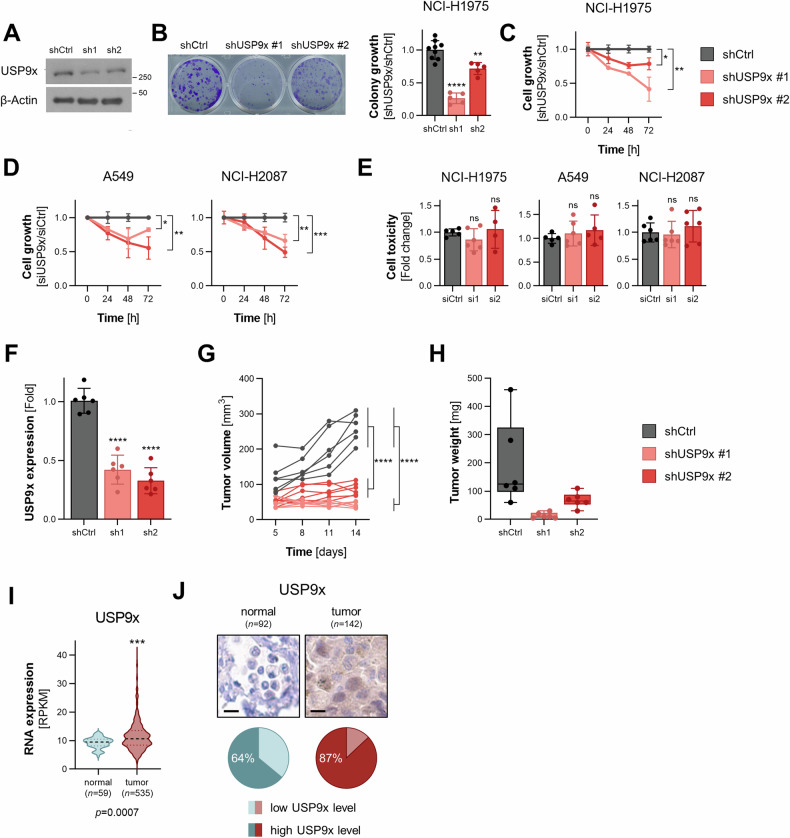
USP9x regulates tumor growth and is elevated in human lung cancer. **A** Knockdown efficiency of stable inducible shRNA-expressing NCI-H1975 cells following induction for 48 h with 20 ng/mL doxycycline. **B** Clonogenicity with quantification (*n* = 3). **C** Cell growth over 4 days upon USP9x knockdown in NCI-H1975 normalized to the non-targeting shRNA control (*n* = 3). **D** Cell growth as in (**C**) in A549 and NCI-H2087 cancer cells upon USP9x knockdown for 48 h (*n* = 3). **E** Measurement of cell toxicity upon USP9x knockdown in the cell line panel (*n* = 3). **F** Knockdown efficiency in xenograft tumors from shRNA-mediated USP9x knockdown cells in mice, (*n* = 6 per condition). **G** Individual tumor growth of USP9x knockdown tumors measured over 14 days. Statistical significance calculated with *t*-tests from the average of each group on day 14. **H** Tumor weight of USP9x knockdown tumors from (**F**). **I** RNA expression of USP9x in lung adenocarcinoma patient samples (*n* = 535) vs. normal lung samples (*n* = 59) obtained from the TCGA database. **J** Representative immunohistochemistry staining for USP9x in lung adenocarcinoma samples (*n* = 142) and normal lung tissue (*n* = 92), the scale bar (in black) measures 10 µm. Error bars ± SD. *p ≤ 0.05, **p ≤ 0.01, ***p ≤ 0.001, ****p ≤ 0.0001.

The guilt-by-association analysis in Fig. 1A predicted that USP9x, beyond being involved in metabolic pathways, was also associated with growth-related activities. To investigate the impact of USP9x on tumor growth, in vitro and in vivo proliferation assays were performed by using doxycycline-inducible shRNA-expressing NCI-H1975 lung cancer cell lines, targeting the USP9x gene or a non-targeting control (Fig. 2A). Using these cells, the clonogenicity and cell growth upon genetic silencing of USP9x in vitro led to a significant growth reduction compared to the control (Fig. 2B, C). Furthermore, the impact of USP9x silencing on cell growth was measured in additional NSCLC cell lines, namely A549 and NCI-H2087, revealing a similar growth reduction as in NCI-H1975 lung cancer cells (Fig. 2D). Importantly, the knockdown of USP9x did not induce cell death in either of the tested lung cancer cell lines (Fig. 2E).

To assess the in vivo effect of USP9x, inducible stable shRNA-expressing NCI-H1975 cell lines were subcutaneously injected into immunodeficient mice, which had continuous access to doxycycline-containing drinking water to ensure constant shRNA expression and downregulation of USP9x. We found that genetic silencing of USP9x resulted in a drastic reduction of tumor growth and tumor weight in vivo (Fig. 2F-H). These results argue that USP9x is critical for lung tumor growth, thus may be selectively elevated in lung cancer tissues compared to non-neoplastic tissue. To test this hypothesis, we undertook two independent approaches. First, the gene expression data of lung adenocarcinoma (LUAD), the largest subgroup of NSCLC, patients from the TCGA uncovered that USP9x transcripts were significantly enriched in lung tumors (*n* = 535) compared to normal lung tissues (*n* = 59; Fig. 2I). Secondly, to evaluate USP9x protein levels in human lung cancer tissues, IHC staining of 142 LUAD samples of all disease stages was performed. By comparing the staining intensities to 92 normal adjacent healthy tissues and normal lung tissues, our IHC analysis revealed that USP9x was specifically expressed in elevated levels in lung cancer tissues (Fig. 2J). While the normal lung tissues displayed 64% of high expression of USP9x, the expression was elevated to 87% in the tumor samples.

Taken together, these data suggest that USP9x is highly expressed in lung cancer tissues and its expression promotes tumor growth.

### USP9x controls the stability of PYCR3

**Fig. 3:**
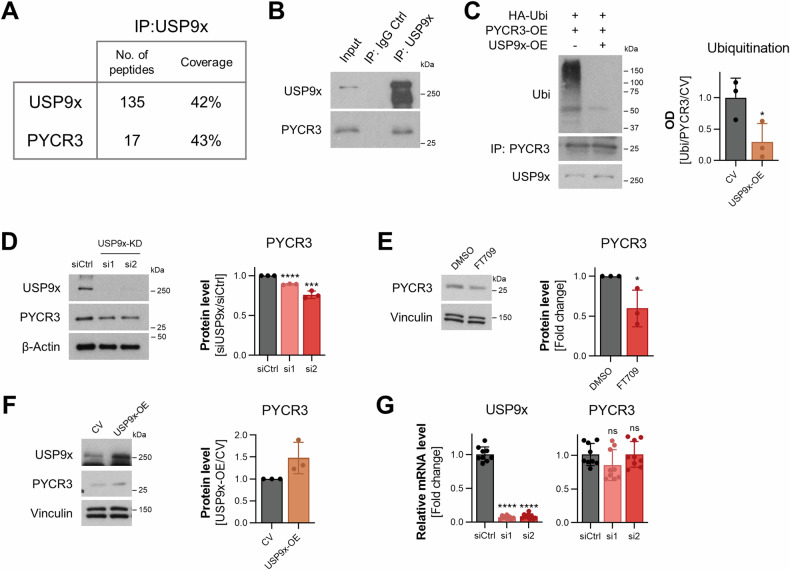
USP9x controls the stability of PYCR3. **A** Number of unique peptides and their coverage obtained in USP9x and PYCR3 from LC-MS/MS analysis on co-immunoprecipitation assay. **B** Co-immunoprecipitates from (**A**) resolved on western blot, normal rabbit IgG was used as control. **C** Ubiquitination assay showing the levels of ubiquitinated PYCR3 in USP9x overexpression vs. control upon treatment with MG132 for 4 h. Bar graph shows quantification of ubiquitinated PYCR3 normalized to total PYCR3 levels and the control. **D** siRNA-mediated knockdown of USP9x, as well as (**E**) USP9x inhibition with the specific inhibitor FT709 for 48 h reduced PYCR3 protein levels. **F** USP9x overexpression elevated PYCR3 protein level. **G** PYCR3 transcription upon USP9x siRNA-mediated knockdown remained unchanged. All quantifications pooled from 3 independent biological replicates. Error bars, ± SD. *p ≤ 0.05, **p ≤ 0.01, ***p ≤ 0.001, ****p ≤ 0.0001.

DUBs target specific proteins and increase their stability through deubiquitination-mediated regulation. Accumulation or simply the reduced clearance of these target proteins can lead to modulations of the pathway activities they are involved in. Some known specific targets for USP9x have been previously identified and are linked to several cellular processes including cell cycle progression by inhibiting the degradation of Cdc20, a component of the mitotic checkpoint complex [28], stress response by stabilizing apoptosis signal-regulating kinase 1 (ASK1) [29], or chromosome alignment by deubiquitinating Survivin [30]. To identify possible metabolic enzymes directly targeted by USP9x, co-immunoprecipitation experiments were performed to elucidate the interaction partners of USP9x. Mass spectrometric analysis of the co-immunoprecipitated proteins revealed several potential interaction partners specific to USP9x (Supplementary Table 2). By focusing on metabolism-regulating enzymes among the identified hits, the pyrroline-5-carboxylate reductase 3 (PYCR3) was identified as a potential interaction partner of USP9x with 17 unique peptides detected, which covered 43% of the protein sequence (Fig. 3A). Immunoblotting further confirmed the presence of this interaction (Fig. 3B).

To test the impact of USP9x on PYCR3 deubiquitination, the deubiquitination activity of USP9x was assessed by treating cells with the proteasomal inhibitor MG132, which prevents the degradation of ubiquitinated proteins. Overexpression of USP9x markedly reduced the level of ubiquitinated PYCR3 compared to the control (Fig. 3C), suggesting that PYCR3 is directly deubiquitinated by USP9x. Next, we examined whether USP9x knockdown or overexpression augments the stability of PYCR3. While both the depletion of USP9x by two independent siRNAs and its inhibition with the selective USP9x inhibitor FT709 reduced PYCR3 protein levels (Fig. 3D, E), USP9x overexpression resulted in elevated PYCR3 protein levels (Fig. 3F). Since knockdown of USP9x did not influence PYCR3 transcription (Fig. 3G), we concluded that PYCR3 is directly regulated by USP9x on the protein level. Hence, we identified PYCR3 as a novel target of USP9x.

### USP9x alters the proline metabolism pathway

**Fig. 4:**
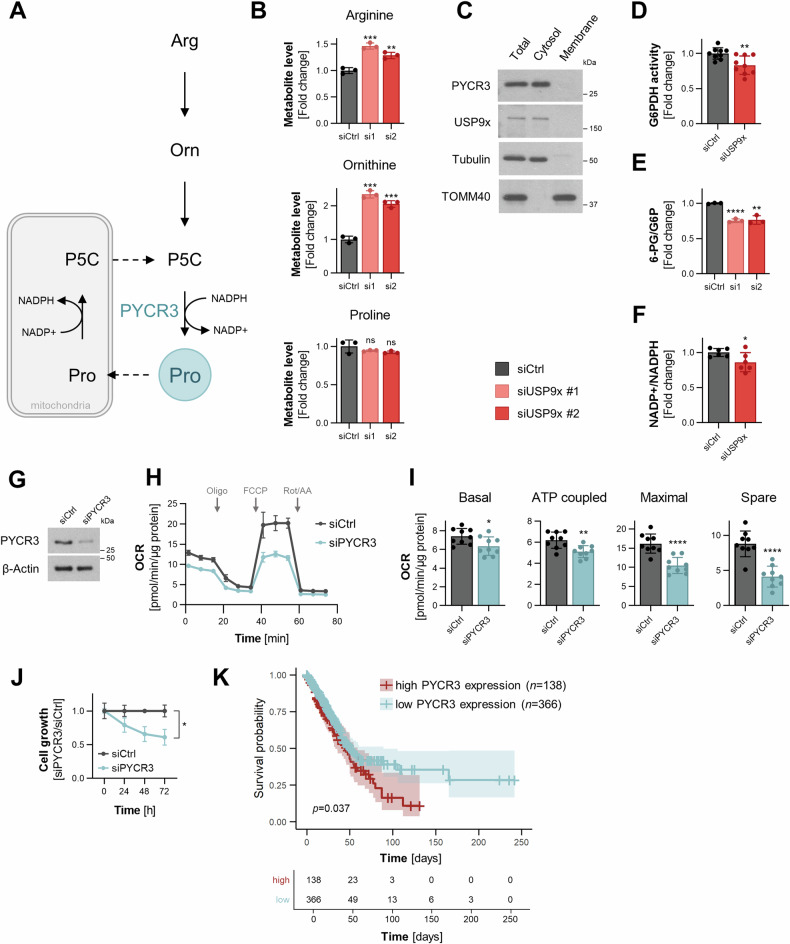
USP9x alters the proline metabolism pathway. **A** Schematic illustration of PYCR3-mediated proline biosynthesis and proline cycle. **B** Metabolite levels of arginine, ornithine, and proline upon USP9x knockdown. **C** Cellular fractionation of NCI-H1975 cells. Tubulin served as a marker for the cytosolic fraction, TOMM40 for the membrane fraction. **D** Enzymatic activity of G6PDH upon USP9x knockdown (*n* = 3). **E** Ratio of metabolite levels of 6-phosphogluconate and glucose-6-phosphate. **F** NADP + /NADPH ratio (*n* = 3). **G** Efficiency of PYCR3 knockdown 48 h post transfection. **H** The effect of PYCR3 knockdown on mitochondrial oxygen consumption rates (OCR) in NCI-H1975 cells normalized to total protein amount. **I** Calculations of indicated mitochondrial parameters from (**H**) (*n* = 3). **J** Cell growth measured over 4 days showing the effect of PYCR3 knockdown in NCI-H1975, normalized to siRNA control. **K** Kaplan–Meier analysis of PYCR3. High expression of PYCR3 is associated with poor overall survival of patients with NSCLC. *P*-value calculated using Log Rank test. Error bars, ±SD. *p ≤ 0.05, **p ≤ 0.01, ***p ≤ 0.001, ****p ≤ 0.0001.

There are three human PYCR isoforms all known to play key roles within proline biosynthesis. The PYCR3 isoform catalyzes the reaction from pyrroline-5-carboxylate (P5C) to proline (Fig. 4A) in the cytosol by utilizing NADPH as a cofactor [31]. In the PYCR3-mediated branch of proline biosynthesis, P5C mainly originates from arginine, ornithine and therefore the urea cycle, while glutamate from the TCA cycle can serve as an alternative source. Our unbiased MSEA of metabolomics data from USP9x depleted cells (Fig. 1C) revealed arginine and proline metabolism among the most significantly altered pathways. Therefore, we focused on individual metabolite levels within the proline biosynthesis pathway to investigate the potential role of USP9x in regulating proline biosynthesis through its effect on the PYCR3 enzyme. Depletion in USP9x led to an accumulation of arginine and ornithine, which are the preferred sources of the PYCR3-mediated proline pathway (Fig. 4B). Minor changes were observed in the alternative sources for the PYCR3-mediated route, namely α-ketoglutarate and glutamate (Supplementary Fig. 1B). Furthermore, proline, the product the proline biosynthesis pathway, was not found to be altered upon USP9x knockdown (Fig. 4B), which is mainly synthesized by PYCR1/2 in the mitochondria. This observation is in line with previous observations that PYCR3 mainly contributes to the proline cycle and not to the overall proline pool within the cell [31]. Cellular fractionation further confirmed that USP9x and PYCR3 are both localized in the cytosol (Fig. 4C). Combined, these results reveal a role for USP9x in the PYCR3-mediated branch of the proline biosynthesis pathway through its regulation of PYCR3.

The proline cycle acts as a redox shuttle between the cytosol and mitochondria transferring NAD + /NADH and NADP + /NADPH to maintain redox homeostasis. It has been reported that recycling of NADP + /NADPH connects proline biosynthesis with the oxidative phase of the pentose phosphate pathway (PPP), contributing to the synthesis of nucleotides [32]. Therefore, we investigated the impact of USP9x depletion on PPP to assess whether its targeting of PYCR3 affects the proline cycle. Indeed, both G6PDH activity and the 6-phosphogluconate/glucose-6-phosphate metabolite ratios were significantly reduced upon genetic silencing of USP9x (Fig. 4D, E), indicating a reduced activity of the NADPH-dependent reactions of the PPP. Furthermore, the ratio of NADP + /NADPH was reduced, suggesting the accumulation of NADPH due to lowered PYCR3 activity upon USP9x knockdown (Fig. 4F). Accordingly, we observed dysregulation of the PPP as well as NADP + /NADPH homeostasis upon USP9x depletion, strengthening our conclusion of USP9x targeting PYCR3 and thus the proline cycle.

In the mitochondrial segment of the proline cycle, proline is degraded to P5C, a reaction catalyzed by proline dehydrogenase (PRODH). In this reaction, PRODH transfers electrons through FADH_2_ to ubiquinone, which is part of the electron transport chain [33]. Therefore, we investigated whether dysregulation of PYCR3 might also affect mitochondrial respiration. Indeed, knockdown of PYCR3 (Fig. 4G) resulted in a significant reduction of mitochondrial respiration (Fig. 4H, I). Moreover, depletion of PYCR3 displayed cell growth retardation in vitro (Fig. 4J). These results suggest a regulatory role of PYCR3 in lung cancer cell growth, likely dependent on its involvement in the proline cycle and its impact on related growth-promoting pathways. We further investigated the potential clinical relevance by examining survival data of lung cancer patients with PYCR3-high *versus* PYCR3-low expression levels. The Kaplan–Meier analysis showed that high expression levels of PYCR3 significantly (*p*-value 0.037) predicted poor prognosis (Fig. 4K). Collectively, these findings suggest that USP9x regulates the proline biosynthesis pathway and the proline cycle via PYCR3, which affects PPP and mitochondrial respiration.

## Discussion

Cancer cells rewire metabolic pathways to supply their energetic and biosynthetic demands for tumorigenesis and rapid proliferation [6]. These metabolic rearrangements are complex and still under investigation. Here, we uncovered a novel aspect of metabolic regulation in lung cancer cells that could offer new avenues for therapeutic interventions.

DUBs are important contributors of healthy protein homeostasis, and their dysregulation has been linked to multiple human diseases highlighting their critical physiological role [16]. Despite many recent efforts, the involvement of DUBs in managing the activity of metabolic pathways in cancer cells is still poorly understood.

The deubiquitinase USP9x has been previously studied in several types of cancer including its involvement in the promotion of liver cancer through modulation of JAK2/STAT3 signaling [34]. In a study on breast cancer, USP9x was found to promote chemoresistance through the stabilization of the transcriptional regulator YAP1 [35]. However, to date, the only known link between USP9x and cellular metabolism has been the fact that USP9x can stabilize mTOR kinase complexes [36, 37], but no direct metabolic target has been identified yet. In this study, we present that USP9x interacts with the PYCR3 enzyme, promoting its stability through deubiquitination-mediated regulation, thus revealing a previously uncovered role of this DUB as a direct regulator of proline metabolism in cancer cells. The disruption of the proline cycle interfered with redox homeostasis and thereby with the PPP and mitochondrial respiration, which are critical for cancer growth.

While the proline cycle has been suggested as a potential therapeutic target in cancer [38], very little is known about its regulation. Kindlin-2 has been shown to be a binding partner of PYCR1 leading to its stabilization in response to the cell mechano-environment [39]. Whether this has implications on the proline cycle or whether Kindlin-2 also targets PYCR3 remains to be further investigated. To our knowledge, no studies have been published yet suggesting how PYCR3 is regulated in cancer so far.

Our study demonstrates USP9x as a direct interactor of PYCR3 preserving enhanced PYCR3 stability. As a result, USP9x activity allows to maintain proline cycling in cancer cells. Given that USP9x depletion significantly affected metabolism in NSCLC cells, warrants this DUB as an important regulator of cancer metabolism. Collectively, our findings provide a novel understanding of the governance of the proline biosynthesis pathway, specifically the PYCR3-mediated branch, in lung cancer cells.

Treatment approaches targeting metabolic vulnerabilities in NSCLC are complicated due to metabolic heterogeneity in cancer patients. Understanding which metabolic profiles exist in lung cancer patients and how these profiles are shaped is important for improving the treatment success. We have previously shown that the DUB JOSD2 is involved in maintenance of serine metabolism in NSCLC and that enhanced serine pathway activity is linked with poor prognosis [40]. Now, our current findings solidify DUBs as important regulators of metabolic rewiring in cancer, warranting DUBs as promising targets for therapeutic intervention of tumor metabolism.

## Materials and methods

### Cell culture

The NSCLC cell lines A549, NCI-H1975, and NCI-H2087 were grown in RPMI-1640 medium (Sigma-Aldrich, St. Louis, MO, USA) supplemented with 10% heat-inactivated FBS (Gibco, Waltham, MA, USA), 2 mM L-glutamine (Sigma-Aldrich), and 100 U/mL penicillin/streptomycin (Sigma-Aldrich). For culturing of the stable Tet-inducible shRNA knockdown cell lines, the medium contained 10% Tet-system-approved FBS (Gibco). The human embryonic kidney cell line HEK293T was cultured in DMEM medium (Gibco) supplemented with 10% heat-inactivated FBS, 2 mM L-glutamine, 100 U/mL penicillin/streptomycin, as well as 1% non-essential amino acids (Sigma-Aldrich), and 100 µM sodium pyruvate (Sigma-Aldrich). All cell lines were kept in a humidified incubator at 37 °C and 5% CO_2_ in a logarithmic growth phase. Frequent PCR-based testing verified the absence of mycoplasma contamination [41, 42].

### Analysis of primary NSCLC patient data

For the guilt-by-association analysis, Spearman correlation was performed between the levels of each of the 99 DUBs used in the screen and the levels of all transcripts, which was computed across all LUAD samples from the TCGA data portal (https://portal.gdc.cancer.gov/). Based on the correlations, the genes were ranked, and used as input for the GSEA analysis of the KEGG pathways using the clusterProfiler package in R [43]. We colored the corresponding DUB-pathway blocks red for highly correlated genes and blue for highly anti-correlated genes, with the color scheme based on the log_10_(*p*-value).

The RNA expression levels of USP9x in LUAD tissues (*n* = 535) were compared to normal lung tissues (*n* = 59). The data was downloaded from the TCGA data portal and visualized as violin plots.

Overall survival of lung cancer patients (*n* = 504) was analyzed by Kaplan–Meier plot with data downloaded from https://kmplot.com/ and based on the RNA expression data from the TCGA. The cutoff to divide patients into PYCR3 expression high and low groups was determined by the performance of all possible cutoff values between the lower and upper quantiles. Plots were generated using the survminer package in R [44].

### Glycolysis screen

Cells were transfected with siRNAs targeting the following DUBs: USP15, FBXO8, USP14, UCK2, USP18, UBL4A, UBL5, STAMBP, UDF1L, UCHL3, COP3S, UCHL5, UCHL1, OTUB2, SENP2, USP1, BRCC3, OTUD6B, UEVLD, USP5, USP10, OTUB1, PSMD14, USP39, and USP9x (ON-TARGETplus^TM^ siRNA pools purchased from Dharmacon, Lafayette, CO, USA). Cells were reseeded into XFe96 cell culture miniplates (6 000 cells/well) 48 h post transfection. On the next day, they were washed and supplied with XF Base medium (Seahorse Bioscience, North Billerica, MA, USA) containing 2 mM L-glutamine, left at 37 °C for 20 min, and then subjected to the assay in an XFe96 Extracellular Flux Analyzer (Seahorse Bioscience). After three baseline measurements, 10 mM glucose and 50 mM 2-deoxyglucose were injected to the cells and three ECAR (extracellular acidification rate) measurements were done after each injection. The obtained ECAR levels were normalized to the protein concentration of each well as measured by BCA assay. Data were plotted as difference in ECAR between DUB knockdown and non-targeting siRNA control.

**Table:** 

Target	Product No.	Target	Product No.
BRCC3	L-005798-00	UCHL5	L-006060-00
COPS5	L-005814-00	UCK2	L-005077-00
FBXO8	L-012435-00	UDF1L	L-017918-00
OTUB1	L-021061-00	UEVLD	L-008494-02
OTUB2	L-010983-00	USP1	L-006061-00
OTUD6B	L-008553-00	USP10	L-006062-00
PSMD14	L-006024-00	USP14	L-006065-00
SENP2	L-006033-00	USP15	L-006066-00
STAMBP	L-012202-00	USP18	L-004236-00
UBL4A	L-020658-00	USP39	L-006087-00
UBL5	L-014320-00	USP5	L-006095-00
UCHL1	L-004309-00	USP9x	L-006099-00
UCHL3	L-006059-00		

### Generation of inducible shRNA-expressing cell lines

For the generation of inducible shRNA-expressing cell lines, shRNA oligomers targeting USP9x (shRNA 1: GAGAGTTTATTCACTGTCTTA, shRNA 2: CGCCTGATTCTTCCAATGAAA) were cloned into the Tet-pLKO-puro plasmid (gift from Dmitri Wiederschain (Addgene plasmid #21915 ; http://n2t.net/addgene:21915 ; RRID:Addgene_21915)) as previously described [45, 46]. A plasmid with a non-targeting randomized shRNA sequence was used for control (gift from Roland Friedel (Addgene plasmid #98398; http://n2t.net/addgene:98398; RRID:Addgene_98398), [47]). Lentiviral particles were produced in HEK293T cells by co-transfecting the cloned shRNA constructs, psPAX2 (gift from Didier Trono (Addgene plasmid #12260; http://n2t.net/addgene:12260; RRID:Addgene_12260)), and pMD2.G (gift from Didier Trono (Addgene plasmid #12259 ; http://n2t.net/addgene:12259 ; RRID:Addgene_12259)) in a 2.5:1.5:1 ratio. The plasmid mixtures were transfected using Lipofectamine^TM^ 2000 (Invitrogen, Thermo Fisher, Waltham, MA, USA) per the manufacturer’s protocol. Medium containing lentiviral particles was collected after 48 and 72 h. NCI-H1975 cells were transduced with the lentivirus-containing medium in the presence of 12 µg/mL polybrene (Sigma-Aldrich) for 24 h. Stable cell lines were generated by selection with 1.5 µg/mL puromycin (Sigma-Aldrich) for 14 days. Knockdown was induced with 20 ng/mL doxycycline (Sigma-Aldrich) for 48 h.

### siRNA-mediated knockdown

Cells were forward transfected using either the Interferin transfection reagent (Polyplus transfection, New York, NY, USA) or the Lipofectamine™ RNAiMAX transfection reagent (Invitrogen, Thermo Fisher) following the recommended manufacturers’ protocols. A non-targeting siRNA served as a control. Knockdown efficiencies were confirmed 48 h post transfection by qPCR or western blot.

**Table:** 

Target		Company	Product No
NT Control Pool		Dharmacon, Lafayette, CO, USA	D-001810-10
USP9x	siRNA #1	Dharmacon, Lafayette, CO, USA	J-006099-06
	siRNA #2	Dharmacon, Lafayette, CO, USA	J-006099-08
PYCR3		Dharmacon, Lafayette, CO, USA	L-014246-00

### Pharmacological inhibition of USP9x

FT709, a specific inhibitor of USP9x [48] was purchased from MedChemExpress (HY-145967, Monmouth Junction, NJ, USA) and prepared according to the manufacturer’s instructions. At approximately 80% confluency, cells were treated with 20 µM FT709 or DMSO only control for 48 h, then collected, lysed and PYCR3 levels were assessed by western blotting.

### Plasmid transfection

A USP9x-coding plasmid vector (RC217531) was purchased from Origene. At 80% confluency, the cells were transfected in 6-well plates with either pCMV empty vector or USP9x plasmid vector using the ViaFect transfection reagent (Promega, Madison, WI, USA) following the manufacturer’s protocol. The transfection mix contained 100 µL of Opti-MEM medium, 2 µg of the plasmid vector and 4 µL of ViaFect per 1 mL of cell culture medium. At 24 and 48 h after transfection, the cells were harvested and lysed for western blotting.

### Western Blot

Cells were lysed using the cOmplete™ Lysis-M buffer (Roche Applied Science, Mannheim, Germany) with protease inhibitors (Roche Diagnostics, Risch-Rotkreutz, Switzerland) and the protein concentrations determined using the Pierce BCA Protein Assay kit (Thermo Fisher Scientific). Samples were mixed with Laemmli sample buffer (BioRad, Hercules, CA, USA), heated, and loaded in equal amounts onto 10% SDS-PAGE. After transfer onto nitrocellulose membranes, they were blocked using 5% non-fat dairy milk in PBS and 0.1% TWEEN® 20 at room temperature. Subsequently, membranes were probed with primary antibodies (diluted in PBS with 1% BSA and 0.1% NaN_3_) overnight at 4 °C. Secondary HRP-conjugated antibodies (goat anti-mouse or goat anti-rabbit, Thermo Fisher Scientific) were diluted in PBS with 2.5% non-fat dairy milk and 0.05% TWEEN® 20. Finally, membranes were developed using Clarity TM Western ECL (BioRad). Protein levels were quantified using the ImageJ software.

**Table:** 

Target	Company	Product no.
β-Actin	Santa Cruz Biotech, Dallas, TX, USA	sc-81178
PYCR3	ProteinTech Group, Chicago, IL, USA	68087-1-Ig
TOMM40	Santa Cruz Biotech, Dallas, TX, USA	sc-11414
Tubulin	Sigma-Aldrich, St. Louis, MO, USA	T8203
Ubiquitin	Cell Signaling Technology, Danvers, MS, USA	3933 S
USP9x	ProteinTech Group, Chicago, IL, USA	55054-1-AP
Vinculin	Abcam, Cambridge, UK	ab129002

### qPCR

RNA was extracted using the PureLink™ RNA Mini Kit (Invitrogen, Thermo Fisher Scientific) and treated with DNase (Thermo Fisher Scientific). For cDNA synthesis, 1 µg of RNA was transcribed using the iScript cDNA synthesis kit (BioRad). The qPCR was performed with cDNA corresponding to 10 ng of total RNA and the Maxima qPCR SYBR green master mix (Thermo Fisher Scientific) and run on the StepOnePlus™ Real-Time PCR System (Applied Biosystems, Waltham, MA, USA). The relative mRNA expression was calculated with the ΔΔC_T_ method, Tubulin was used as the housekeeping gene.

**Table:** 

Target		Sequence
PYCR3	fw	5’-GCCCCCAAACACACGGG-3’
	rv	5’-CCATCACTATGGCCCCTTCCT-3’
Tubulin	fw	5’-TCTACCTCCCTCACTCAGCT-3’
	rv	5’-CCAGAGTCAGGGGTGTTCAT-3’
USP9x	fw	5’-GTGTCAGTTCGTCTTGCTCAGC-3’
	rv	5’-GCTGTAACGACCCACATCCTGA-3’

### In vitro growth assays

After inducing the knockdown with either doxycycline in the stable shRNA-expressing cell lines or performing the transfection with siRNA, cells were reseeded in triplicates in a 96-well plate at 1 000 – 2 000 cells/well. Cell growth was assessed for 4 days using the CellTiter-Glo® luminescent cell viability assay (Promega, Madison, WI, USA). The signal was read on a GloMax Discover Microplate Reader (Promega) and the values normalized to the 0 h time point and to the respective controls.

For the clonogenic growth assay, cells were reseeded after induction of the knockdown with doxycycline for 48 h at 1 000 cells/well in 12-well plates. Medium was replaced every 72–96 h and the cells cultured for a total of 7–9 days. Cells were then washed with PBS, fixed with 1% paraformaldehyde (Sigma-Aldrich), and stained with 0.1% crystal violet (Sigma-Aldrich). For quantification, each well was treated with 200 µL 1 M NaOH, 2 × 75 µL transferred into a black 96-well plate, and the solution neutralized with 1 M HCl. Fluorescence was detected using the GloMax Discover Microplate Reader, with an excitation wavelength of 520 nm and emission wavelength of 580–640 nm. The background fluorescence was subtracted, and the values normalized to the control condition.

### Cell toxicity

Cell toxicity after siRNA-mediated knockdown was determined using the bioluminescent ToxiLight^TM^ BioAssay Kit (Lonza, Basel, Switzerland) using the manufacturer’s protocol. Cell toxicity was measured after 48 and 96 h post transfection and the values normalized to the former time point.

### Animal studies

For the in vivo studies, female 4–5 week old immunodeficient Athymic nude mice (Rj:ATHYM-Foxn1nu/nu*) were purchased from Janvier Labs (Le Genest-Saint-Isle, France). Mice were housed in pathogen-free conditions at the Karolinska Institute animal facility with temperatures between 20–24 °C and a 12 h light/dark cycle. All experiments were performed in accordance with the Swedish animal welfare laws authorized by the Stockholm Animal Ethics Committee. For the tumor growth study, 18 mice were randomly divided into 3 groups, each group consisting of 6 mice prior to injection of shRNA-expressing cells.

### In vivo tumor growth

Stable shRNA-expressing NCI-H1975 cells were treated with 20 ng/mL doxycycline for 48 h to ensure homogenous induction of the knockdown prior the subcutaneous injection of 3 × 10^6^ cells/mouse in 100 µL of 1:1 PBS and Matrigel (Corning Inc., Corning, NY, USA) into the flanks. During the experiment, mice were provided with doxycycline-containing drinking water (2 mg/mL doxycycline, supplemented with 5% sucrose (Sigma-Aldrich)). To track the tumor volume, tumors were measured with a caliper every third day. At the end of the experiment, the mice were sacrificed, the tumors were removed, weighed, frozen in liquid nitrogen, and stored at -80 °C until further analysis. To calculate the tumor volume, the following formula was used: 2 W x L/2 (L = length and W = the perpendicular width of the tumor, L > W). The knockdown efficiency of the tumors was validated by qPCR.

### Immunohistochemistry

Immunohistochemistry staining for USP9x was performed using the Autostainer 480 (Thermo Fisher Scientific, Waltham, MA, USA) as previously described [49]. Briefly, tissue slides were incubated in Ultra V block (Thermo Fisher Scientific) for 5 min and stained with an antibody targeting USP9x (CAB070164, Human Protein Atlas) for 30 min. Afterwards, slides were incubated with HRP polymer for 30 min, with 3,3′-diaminobenzidine solution for 5 min, counterstained in Mayers hematoxylin for 5 min using the Autostainer XL (Leica Biosystems, Nussloch, Germany), and finally they were rinsed in lithium carbonate water for 1 min. The slides were dehydrated in graded ethanol and coverslipped (PERTEX, Histolab) using the CV5030 automated glass coverslipper (Leica Biosystems, Nussloch, Germany). The slides were scanned using the Aperio XT automated scanning system (Leica Biosystems, Nussloch, Germany).

The non-neoplastic tissues included normal lung, adjacent lung, and matched adjacent lung tissues (*n* = 92). All tumor tissues were lung adenocarcinoma (*n* = 142). The following scoring criteria were used for the immunohistochemical stainings as analyzed with ImageJ; negative (0), weak (1), moderate (2), and strong [3]. USP9x-high includes strong (3) and USP9x-low includes (2) down to (0).

### Co-immunoprecipitation

For co-immunoprecipitation experiments, NCI-H1975 cells were seeded in 150 mm^2^ dishes (3 dishes per condition) and collected the next day by scraping. The cells were pelleted by centrifugation and lysed in lysis buffer from the MS/MS kit (Pierce™ MS-Compatible Magnetic IP Kit, Thermo Fisher) for 10 min on ice. After centrifugation (13 000 *g*, 5 min), the supernatant was collected, the protein concentration was measured, and the samples equally divided into several tubes including isotype antibody control (Abctr5 Proteinlab or Abctr6 Thermo Fisher) and USP9x.

20 µL of the Protein A/G Magnetic Beads (Pierce™ MS-Compatible Magnetic IP Kit, Thermo Fisher) were conjugated with either the isotype antibody control or the USP9x antibody (1.5 µg each) in lysis buffer for 6 h at 4 °C on a rotating platform. Then, the antibody-bead complexes were washed once with the lysis buffer and incubated overnight with the cell lysates. The beads were washed according to the kit protocol. 10% of the bead-containing protein complexes was used for immunoblotting and the remaining part (corresponding to 0.6 mg of initial total protein lysate) was sent for the LC-MS/MS analysis.

### Mass spectrometric analysis of immunoconjugates

Mass spectrometric analysis was performed by the Proteomics Biomedicum Core Facility at the Karolinska Institute. Immunoconjugates were digested with trypsin with the complexes remaining on the magnetic beads. Peptides were purified and resuspended in 2% acetonitrile (ACN), 0.1% formic acid (FA) in a total volume of 15 µL. For the Nano-flow LC-MS/MS analysis, 2 µL of the samples were injected into the Ultimate 3000 UHPLC chromatographic system (Thermo Fisher Scientific) coupled on-line to a Q Exactive™ HF hybrid Quadrupole-Orbitrap™ mass spectrometer (Thermo Fisher Scientific). Separation of the peptides was done on a 50 cm long heated (55 °C) C-18 Easy-Spray™ column (Thermo Fisher Scientific) using an organic gradient 4–36% B in 120 min at 300 nL/min flow rate (solvent A: 2% ACN, 0.1% FA; solvent B: 98% ACN, 0.1% FA). Survey mass spectra were acquired at mass resolution of 120 000 (range of m/z 350-600). MS/MS data of the 17 most intensive precursors were obtained at a resolution of 30 000 by higher-energy collisional dissociation (HCD) with 28% normalized collision energy. The in-house Raw2mgf program was used to convert the MS raw data files into Mascot Generic Format (mgf). The mgf files were searched against the SwissProt database (HUMAN) with the Mascot Server search engine (v2.5.1, MatrixScience Ltd., London, UK) allowing up to two missed cleavage sites for trypsin, a mass tolerance of 10 ppm, and 0.02 Da for the precursors and HCD fragment ions, respectively. Cysteine carbamidoethylation was used as fixed modification, while deaminations of asparagine and glutamine, as well as oxidation of methionine were dynamic. Proteins with any number of detected unique peptides in the USP9x conjugates and no peptides in both controls were considered as potential interaction partners.

### Ubiquitination assay

NCI-H1975 cells were co-transfected with overexpression plasmids using the above-described procedure and using the following constructs: Control Vector (pCB6 + )/USP9x overexpression plasmid (RC217531, Origene) + PYCR3 overexpression plasmid (RC203382, Origene) + HA-Ubiquitin overexpression vector (gift from Edward Yeh (Addgene plasmid #18712; http://n2t.net/addgene:18712; RRID:Addgene_18712), [50]). At 24 h later, cells were treated with 5 µM of the proteasomal inhibitor MG132 (Selleckchem, Houston, TX, USA) for 4 h prior harvesting. The lysates were used for immunoprecipitation with the antibody against PYCR3. Proteins were resolved by western blotting on a 12% SDS-PAGE. Immunoblotting with anti-Ubiquitin and anti-PYCR3 antibodies was performed to assess the ubiquitination status PYCR3 upon USP9x overexpression. Level of ubiquitinated PYCR3 was quantified using ImageJ.

### Metabolomics

At 48 h post transfection with knockdown-inducing siRNAs, metabolites were extracted from the cells as previously described [51]. Briefly, cells were washed with 5% mannitol (Sigma-Aldrich) solution and metabolites extracted with methanol (Sigma-Aldrich) containing 10 µM Internal Standard Solution (Human Metabolome Technologies, Boston, MA, USA). The extracted metabolites were centrifuged at 2 300 *g* at 4 °C for 5 min and the supernatant filtered through a Millipore 5-kDa cutoff filter at 9 100 *g* at 4 °C for 2 h. The filtrate was analyzed using Capillary-Electrophoresis Time of Flight Mass Spectrometer (CE-TOF/MS, Human Metabolome Technologies).

### Metabolite Set Enrichment Analysis (MSEA)

For the metabolite set enrichment analysis (MSEA) the MetaboAnalystR package was used. Dot plots were used to represent the pathways that were significantly impacted by knockdown of both siRNAs, with the color and size of the dots representing the -log_10_ of the corrected *p*-value and the number of metabolites that were enriched in each pathway, respectively.

### Sub-cellular fractionation

The NCI-H1975 cells were seeded on 100 mm^2^ dishes one day before the experiment. The cells were washed twice with PBS and collected using cell scrapers. The cell suspension was equally divided among two tubes to prepare total lysate and cellular fractions. The total lysates were prepared by lysing cells for 15 min on ice in RIPA buffer, supplemented with cOmplete^TM^ protease and phosphatase inhibitor cocktails. The cytosolic fraction was prepared by lysing cells in buffer A (150 mM NaCl, 50 mM Hepes, pH 7.4, 0.02% digitonin, cOmplete^TM^ protease and phosphatase inhibitor cocktails) and incubated for 5 min at room temperature. The obtained extract was centrifuged at 15 000 *g* for 10 min and the supernatant was collected as the cytosolic fraction. Then, the pellets were washed 3 times with PBS and the membrane fraction containing mitochondria was extracted using buffer B (RIPA lysis buffer supplemented with cOmplete^TM^ protease and phosphatase inhibitor cocktails) for 15 min on ice. After centrifugation (15 000 *g*, 10 min), the supernatant was collected as the fraction containing mitochondria. TOMM40 served as a marker for the membrane fraction, and Tubulin for the cytosolic fraction.

### G6PDH activity assay

The G6PDH enzymatic activity was determined using the colorimetric assay kit by Abcam (ab102529) and following the manufacturer’s instructions. Briefly, cells were harvested 48 h post transfection with siRNAs for USP9x. After centrifugation at 12 000 *g* for 5 min at 4 °C, the supernatant was collected for subsequent analysis. The reaction mix was added according to the protocol and the absorbance measured after 6 min at 450 nm wavelength on the GloMax Discover Microplate Reader (Promega) at 37 °C. The enzymatic activities were normalized to protein concentrations and to the control condition.

### NADP + /NADPH assay

NADP+ and NADPH levels were determined using the bioluminescent NADP/NADPH-Glo^TM^ Assay by Promega (G9081). Briefly, cells were lysed in base solution with 1% DTAB (Sigma-Aldrich). The cell lysates were split into two parts to enrich for NADP+ and NADPH by acid and base treatment, respectively. To measure NADP + , the samples were treated with 0.4 N HCl and heat quenched at 60 °C for 15 min, while the NADPH samples were heat quenched in the 1% DTAB base solution. The samples were neutralized with Trizma or HCl-Trizma after cooling down to room temperature. The treated cell lysates were mixed in equal parts with the NADP/NADPH-Glo^TM^ detection reagent, incubated for 45 min, and the luminescence measured with the GloMax Discover Microplate Reader.

### Determination of mitochondrial respiration

To determine mitochondrial respiration, the oxygen consumption rate (OCR) was measured in real-time using the XFp Extracellular Flux Analyzer (Seahorse Bioscience). After induction of the knockdown using siRNA transfection for 48 h, cells were reseeded in triplicates at a density of 6 000 cells/well in an XFp miniplate. On the next day, cells were washed, and the medium changed to XF Base medium (Seahorse Bioscience) with 1 mM pyruvate, 2 mM glutamine, and 10 mM glucose about 30 min before the start of the assay. After a baseline measurement, 1 μM oligomycin, 0.5 µM FCCP, and 0.5 µM rotenone/antimycin A were injected to the cells over the course of approximately 1.5 h.

### Statistical analysis

All graphs were designed, and the statistics calculated using the GraphPad Prism Software version 9.0.0 (Dotmatics, Boston, MA, USA) or R version 4.2.0. The data were presented as the mean ± S.D. Unless otherwise mentioned, unpaired *t*-tests were used for statistical analysis. The specifics for each panel are described in the respective figure legend. *p ≤ 0.05, **p ≤ 0.01, ***p ≤ 0.001, ****p ≤ 0.0001.

### Supplementary information

Supplementary Figure_Table_Legends

Supplementary Figure 1

Supplementary Table 1

Supplementary Table 2

Original Western-blotting

## Data Availability

The datasets generated during and/or analyzed during the current study are available from the corresponding author on reasonable request.
