# Supplementary material for: Deubiquitinase USP9x regulates the proline biosynthesis pathway in non-small cell lung cancer
Source: Cell Death Discov. 2024 Jul 29;10:342. doi: 10.1038/s41420-024-02111-2 (PMC11286954; doi:10.1038/s41420-024-02111-2)
Supplement: Supplementary file 1 — Supplementary Figure_Table_Legends [file 41420_2024_2111_MOESM1_ESM.docx]

**Supplementary Fig. 1**

**A** Knockdown efficiency of USP9x used for metabolomics analysis. **B** Metabolite levels of glutamate and α-ketoglutarate upon USP9x knockdown from (A). Error bars, ± SD. * *p* ≤ 0.05, ** *p* ≤ 0.01

**Supplementary Table 1**

Results of metabolite set enrichment analysis from metabolite levels measured in USP9x-deficient cells compared to cells expressing the non-targeting control. Only pathways with a significant FDR (<0.05) are listed in the table.

**Supplementary Table 2**

USP9x-associated proteins investigated by co-immunoprecipitation followed by LC-MS/MS analysis. Proteins with no detected peptides in the control antibodies and at least one peptide detected in the USP9x immunoconjugates are listed in the table.
