# Supplementary figures and images for: Deubiquitinase USP9x regulates the proline biosynthesis pathway in non-small cell lung cancer
Source: Cell Death Discov. 2024 Jul 29;10:342. doi: 10.1038/s41420-024-02111-2 (PMC11286954; doi:10.1038/s41420-024-02111-2)

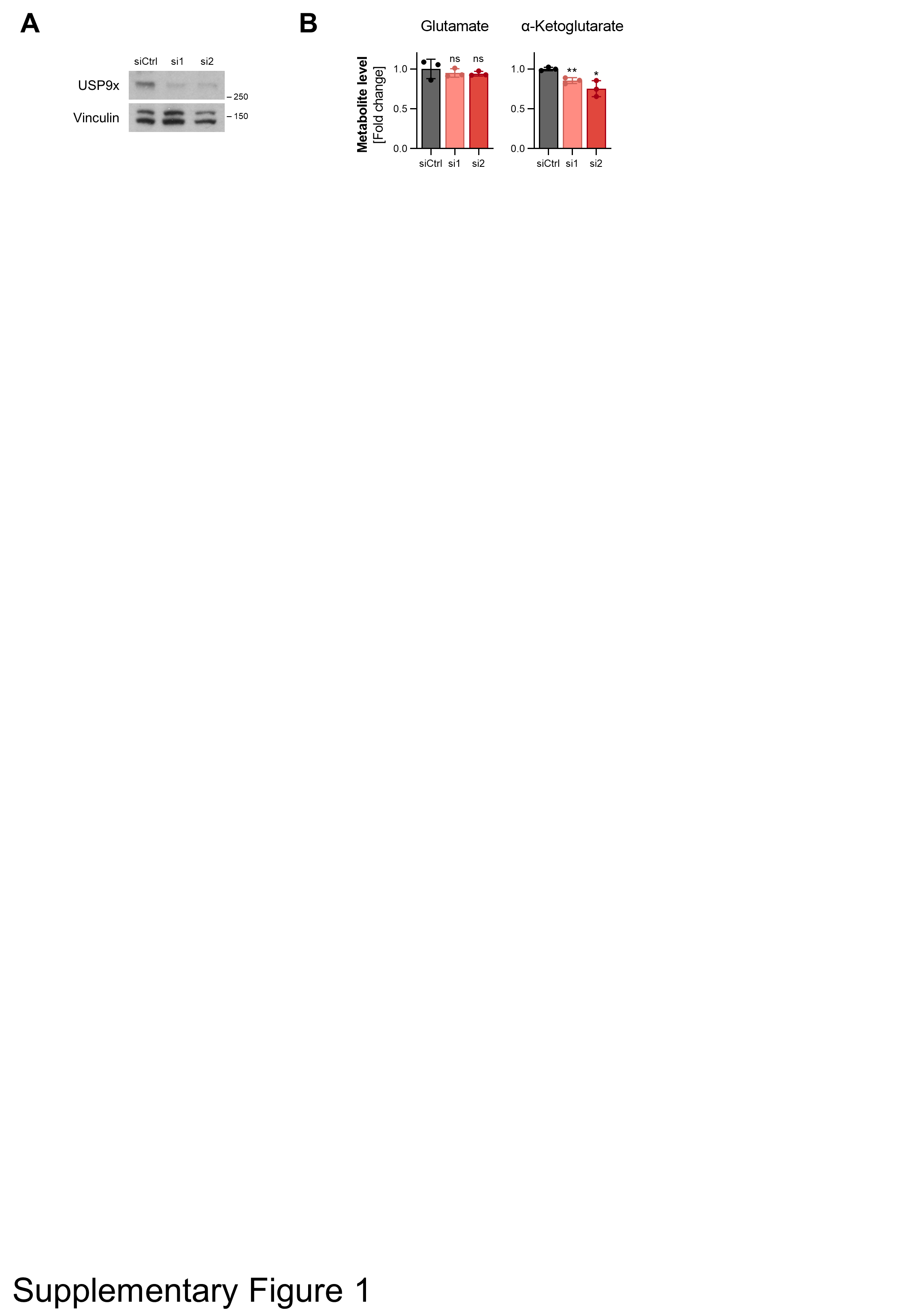

Supplement: Supplementary file 2 — Supplementary Figure 1 [file 41420_2024_2111_MOESM2_ESM.tif]

Fig. 2A

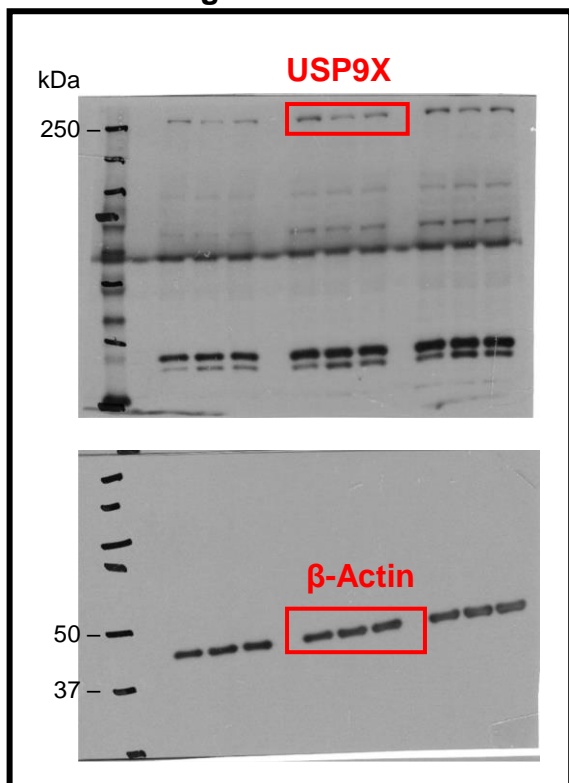

Fig. 3B

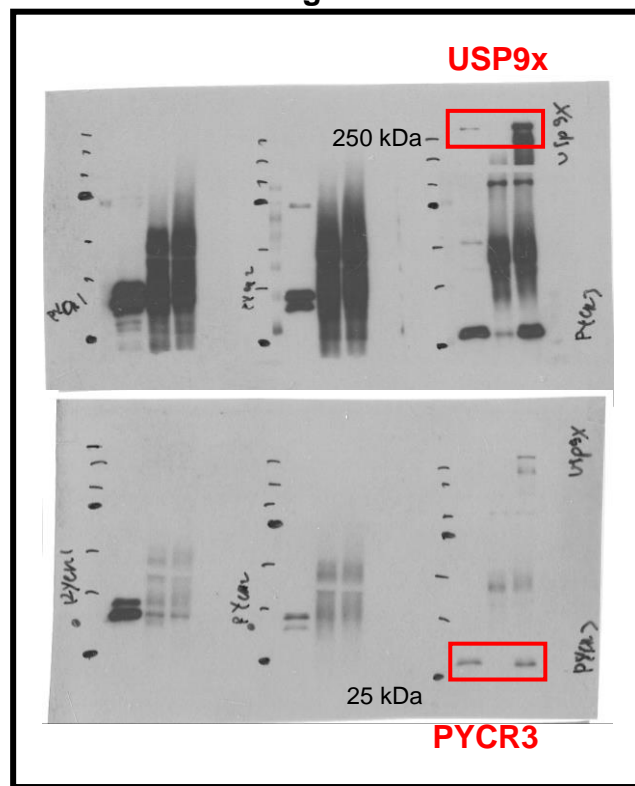

Fig. 3C

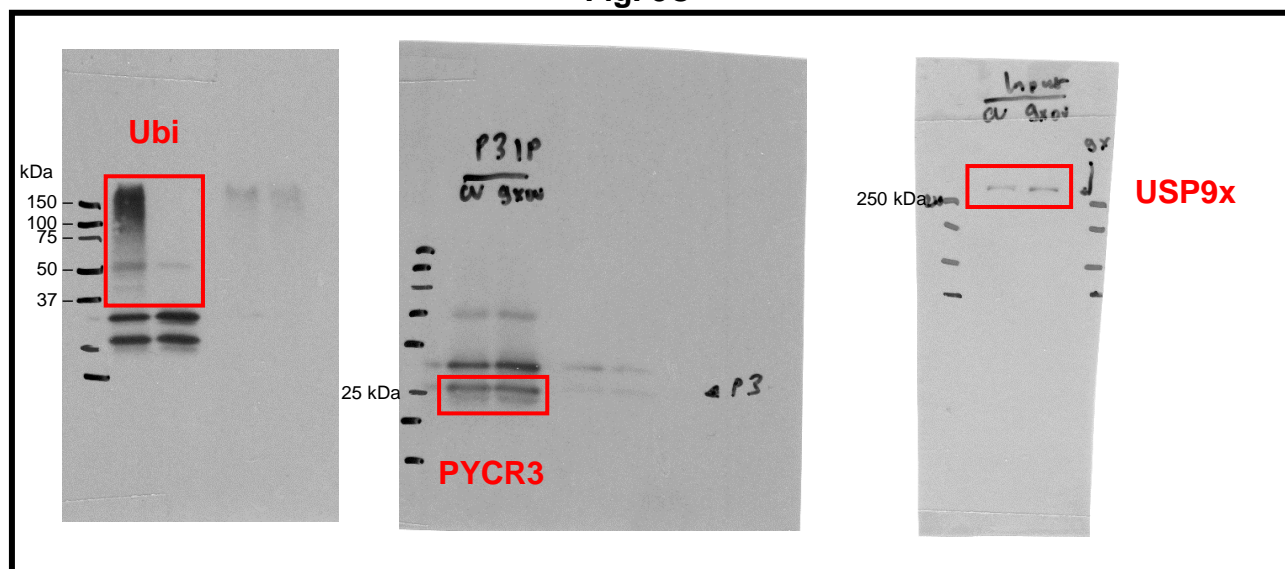

Fig. 3D

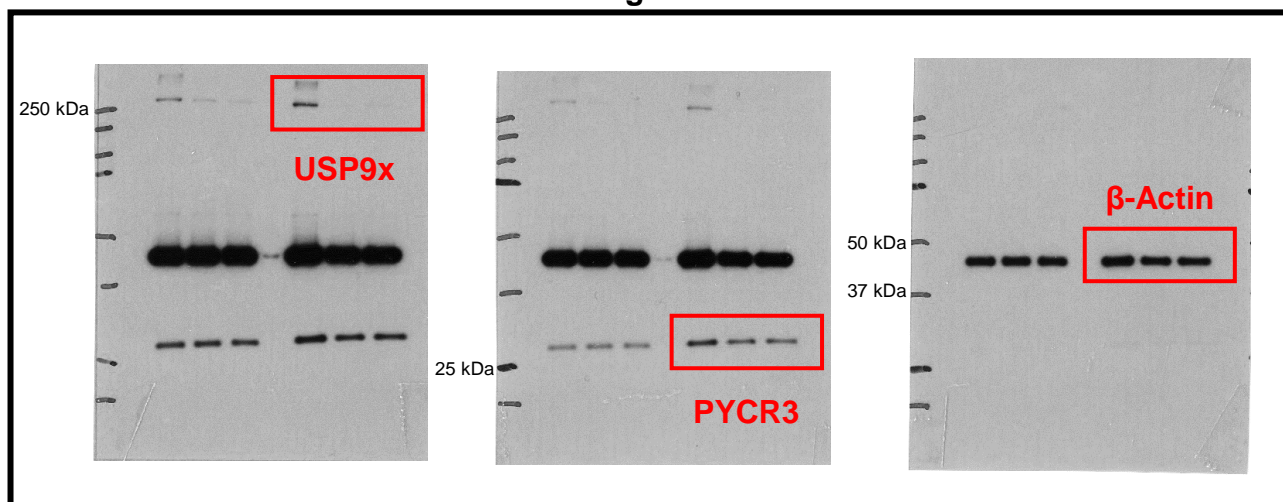

**Fig. 3E**

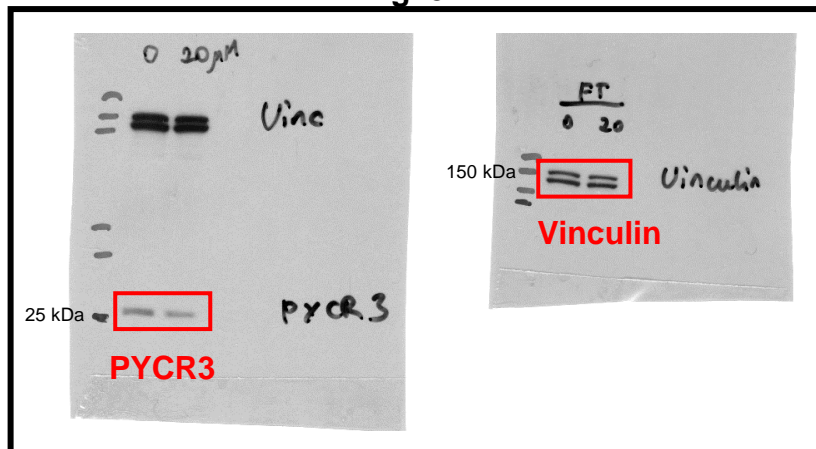

**Fig. 3F**

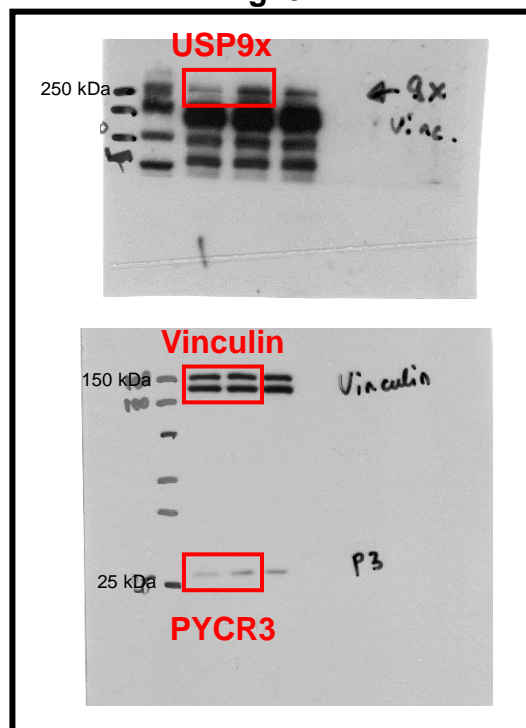

**Fig. 4C**

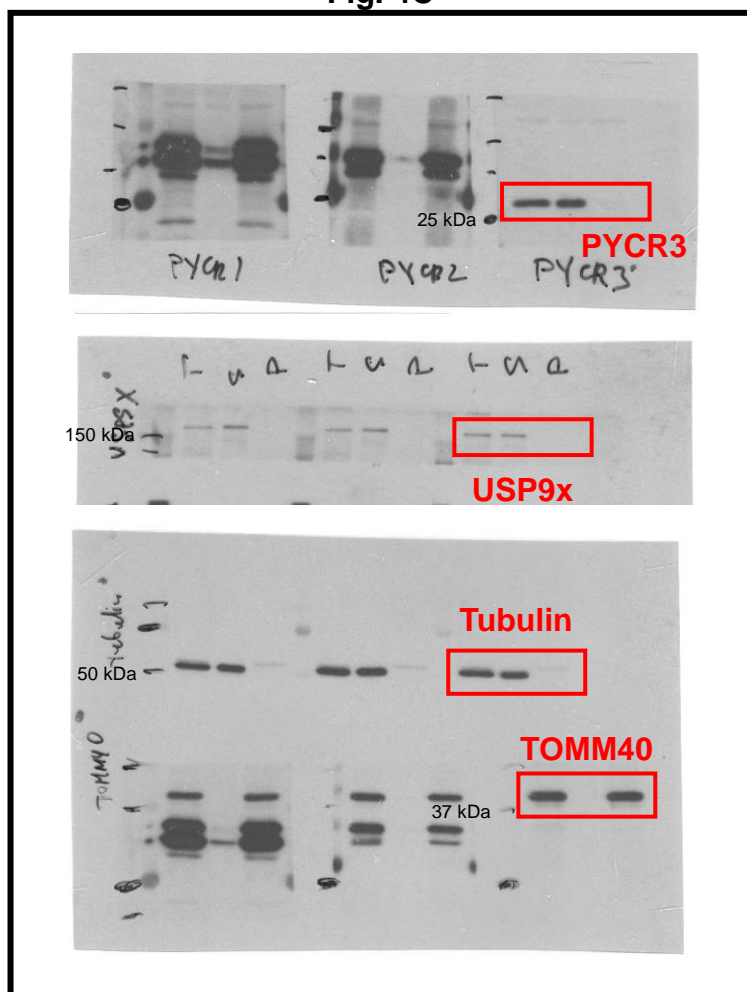

**Fig. 4G**

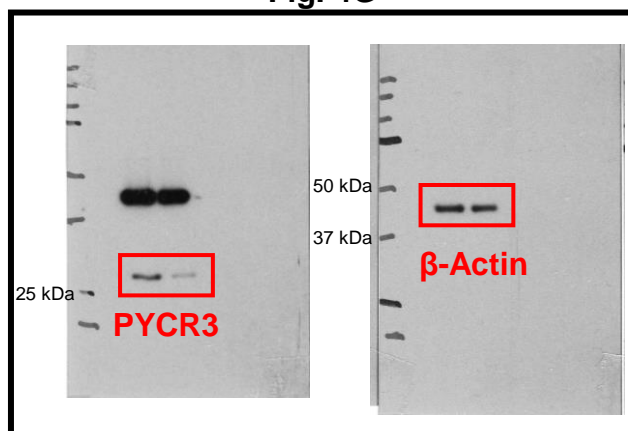

**Supplementary Fig. 1A**

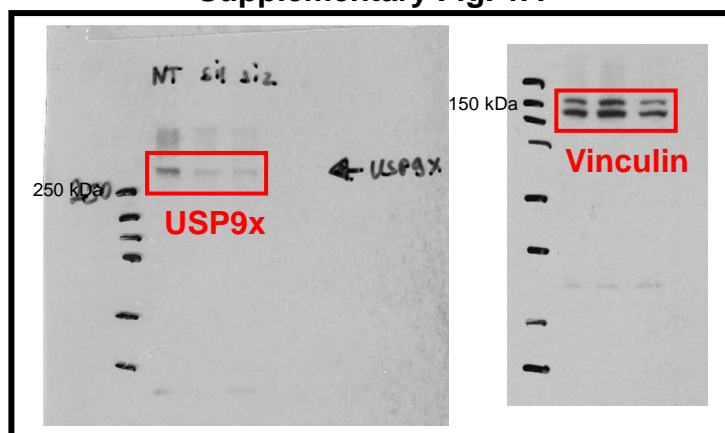

Supplement: Supplementary file 5 — Original Western-blotting [file 41420_2024_2111_MOESM5_ESM.pdf]
